# A data mining approach for grouping and analyzing trajectories of care using claim data: the example of breast cancer

**DOI:** 10.1186/1472-6947-13-130

**Published:** 2013-11-30

**Authors:** Nicolas Jay, Gilles Nuemi, Maryse Gadreau, Catherine Quantin

**Affiliations:** 1 Université de Lorraine, LORIA UMR 7503, F-54000, Nancy, France; 2 CHU de Nancy, Département d’information médicale, F-54000, Nancy, France; 3 CHRU Dijon, Service de Biostatistique et d’Informatique Médicale (DIM), F-21000, Dijon, France; 4 Inserm, U866, Univ de Bourgogne, F-21000, Dijon, France; 5 Université de Bourgogne, F-21000, Dijon, France

**Keywords:** Data mining, Formal concept analysis, Claim data, Trajectory of care, Cancer

## Abstract

**Background:**

With the increasing burden of chronic diseases, analyzing and understanding trajectories of care is essential for efficient planning and fair allocation of resources. We propose an approach based on mining claim data to support the exploration of trajectories of care.

**Methods:**

A clustering of trajectories of care for breast cancer was performed with Formal Concept Analysis. We exported Data from the French national casemix system, covering all inpatient admissions in the country. Patients admitted for breast cancer surgery in 2009 were selected and their trajectory of care was recomposed with all hospitalizations occuring within one year after surgery. The main diagnoses of hospitalizations were used to produce morbidity profiles. Cumulative hospital costs were computed for each profile.

**Results:**

57,552 patients were automatically grouped into 19 classes. The resulting profiles were clinically meaningful and economically relevant. The mean cost per trajectory was 9,600€. Severe conditions were generally associated with higher costs. The lowest costs (6,957€) were observed for patients with in situ carcinoma of the breast, the highest for patients hospitalized for palliative care (26,139€).

**Conclusions:**

Formal Concept Analysis can be applied on claim data to produce an automatic classification of care trajectories. This flexible approach takes advantages of routinely collected data and can be used to setup cost-of-illness studies.

## Background

Health-care systems face a crisis of an increasing burden of chronic diseases aggravated by aging populations [1]. It is of much importance that policy makers and healthcare managers can make decisions based on sufficient knowledge and understanding of chronic care activities. This is especially true in the field of cancer where incidence, therapeutics, practices and costs can vary quickly [2,3]. On the one hand, policy-makers need cost-effectiveness and cost-of-illness analyzes for planning and fair allocation of funding. On the other hand, care providers should be able to adapt their resources and costs while they share patients in multidisciplinary and coordinated approaches. Costs can be estimated from a variety of data sources, including insurance claims, billing systems, hospital discharge databases and surveys [4]. However, data sources may vary in a number of important aspects: accessibility, representativeness, level of aggregation, period of observation, availability and accuracy of clinical data. Besides, discrepancies can be observed depending on the source used to identify cases or estimate medical expenditures [5].

In parallel to ad hoc surveys that are often temporary and costly, administrative data are routinely collected in perennial information systems. They are an easily accessible source of information to analyze the economical burden of chronic diseases [6]. Moreover, when they contain enough clinical details, claim databases have proven to be useful in the field of epidemiology [7-12]. Though essentially used for funding and analysis of isolated episodes of care, this combination of medical and economical information may contain sufficient ingredients to study trajectories of care, giving better and more comprehensive insights on the journey of chronic patients in the healthcare system [13].

In France, the *Programme de Médicalisation des Systèmes d’Information * (PMSI) is a nationwide information system, derived from the Diagnosis Related Groups (DRG) system [14]. Initially build for billing purposes, the PMSI system has two important advantages for the analysis of trajectories of care. Each sector of activity (acute, post-acute, psychiatric care) is covered by an information system common to the whole French population. Second, since the introduction of an anonymised identifier in 2001, it allows the linkage of all hospitalizations of a same patient across time, space and sectors of activity. However, as most of existing patient classification systems, the PMSI focuses on single contacts and was not designed to categorize a care process spanning several encounters. For chronic conditions, it is of much interest to summarize the information contained in a set a longitudinal data and produce meaningful categories that will be relevant for subsequent analysis. This is a difficult and time consuming classification task, as chronic patients can have multiple diagnoses and multiple treatments recorded in several different facilities, and because it is an indirect and a posteriori use of data that were initially collected for budgetary purposes. Besides, different classifications may be required to achieve different goals. Meanwhile, data mining methods may support the experts in the categorization and analysis of trajectories of care [15].

In this article, we propose a method for grouping trajectories of care over a sequence of hospitalizations, using claim data. Our approach relies upon Formal Concept Analysis (FCA), a conceptual clustering method, and data from the PMSI. We studied one-year trajectories of care of the patients having undergone breast cancer surgery in 2009 in France.

## Methods

### Formal concept analysis

Introduced by Wille [16], Formal Concept Analysis (FCA) is a theory of data analysis identifying conceptual structures within data sets [17]. FCA is closely related to the well-known Association Rule Mining (ARM) and frequent itemsets discovery methods [18]. Indeed, many of the most efficient ARM algorithms are FCA-based [19-21]. A key advantage of the FCA-like mining lays in the fact that due to closure properties, only patterns of maximal size are extracted. This ability to produce condensed representation of patterns or rules reduces the exploration/interpretation burden for the analyst [22]. Another strong feature of FCA is its capability of discovering inherent hierarchical structures within data and thereby producing graphical visualizations. FCA has been successfully used in various health related applications [23-28].

FCA mathematizes the philosophical understanding of a concept as a knowledge unit consisting of two parts: the extent and the intent. The extent covers all objects (or entities) that are instances of the concept, while the intent comprises all attributes (or properties) holding for all the objects under consideration. FCA starts with a formal context defined as a triple *K* = (*G*,*M*,*I*) where *G* is a set of objects, *M* a set of attributes and *I* a binary relation between *G* and *M*. (*g*,*m*) ∈ *I* means that the object *g* has the attribute *m*. *K* may be seen as a table relating objects and their attributes. The Table 1 shows a formal context *K* representing the relation *I* between a set of 8 objects *G* = {1,…,8} and a set of 4 attributes *M* = {*a*,*b*,*c*,*d*}. A cross indicates that a given object has the corresponding attribute. Two operators both denoted by ^′^ can be defined on the power sets of objects 2^*G*^ and attributes 2^*M*^ as follows: 

**Table 1:** **A formal context *K***

	**a**	**b**	**c**	**d**
1	X			
2		X	X	
3		X		X
4		X	X	
5	X			
6	X	X	X	
7			X	
8		X		X

$${\;}^{\prime }:2^{G}\to 2^{M},X^{\prime }=\{m\in M|\forall g\in X,(g,m)\in I\}$$

The ^′^ operator is dually defined on attributes. A formal concept of *K* is a pair (*A*,*B*) with *A* ⊆ *G* and *B* ⊆ *M* such that *A*^′^ = *B* and *B*^′^ = *A*. *A* is called the extent and *B* is called the intent of the formal concept. For example, *C* = ({*g*2,*g*4},{*m*1,*m*2}) is a formal concept of *K*. A subconcept - superconcept relation can be formalized as:

$$(A_{1},B_{1})\leq (A_{2},B_{2})\Leftrightarrow A_{1}\subseteq A_{2}\Leftrightarrow B_{2}\subseteq B_{1}$$

**Figure 1:**
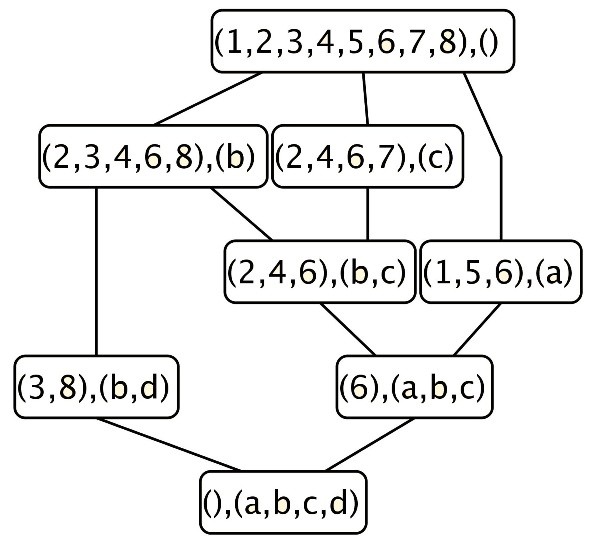
**Concept lattice of the formal context *K*.**

The set of all concepts of a formal context *K* = (*G*,*M*,*I*) together with the order relation form a complete lattice and can be displayed in a line diagram as shown in the right part of Figure 1 for the formal context of Table 1. Such diagrams can be very useful in the field of knowledge discovery to understand conceptual relationships among data. However, the number of formal concepts increases, at worst exponentially, with the size of the formal context. Some interest measures for concepts have been proposed to reduce the complexity of concept lattices [29-31]. The *support* of a concept (*A*,*B*) is the number of objects in its extent [29]: 

support(A,B)=|A|

Among these objects, some may have exactly the properties of the intent and no more. Such objects are called the own objects of a concept. Formally, given a concept (*A*,*B*), the set of own objects of this concept is:

$$\left\{g\in A|{\left\{g\right\}}^{\prime }=B\right\}$$

For example, in Figure 1, 7 is an own object of the concept {2,4,6,7},{*c*}. The concept {2,3,6,8},{*b*} has no own object because 3 and 8 share also *d* and 2, 4, 6 share also *c*. The proportion of own objects in a concept is a measure of the cohesion of its objects. When this proportion is low, the objects of the concept are likely to have a more specific description and to also appear in its subconcepts. The concept may then be seen as not comprehensive enough. Noise in data tends to generate such concepts. In our experiment, we have filtered the lattice by discarding concepts with low support and low proportion of own objects.

### The PMSI database

The PMSI is the French casemix system database. It is mandatory in all public and private hospitals where each admission triggers the collection of a minimal set of data holding administrative and clinical information. The PMSI database hold 24,575,239 stays in 2009. The coding of diagnoses and procedures forms the basis of information needed for the definition of patient groups. Diagnoses are coded with the 10^th^ International Classification of Diseases (ICD-10) and medical procedures with the french nomenclature “Classification Commune des Actes Médicaux (CCAM)”. In the PMSI, diagnoses can have different roles: principal, related or comorbidity. The principal diagnosis is the condition problem that is chiefly responsible for occasioning the admission of the patient to the hospital for care. When it is chosen in Chapter XXI (Factors influencing health status and contact with health services), the principal diagnosis may be precised by a related diagnosis giving the aetiology. Table 2 gives an example of an inpatient record in the PMSI database.

**Table 2:** A PMSI record

Patient ID	XXXXX
Hospital id	54000278
Stay index	12235
Age	56
Gender	F
Admission month	3
Admission year	2009
Admission status	home
Stay duration	10
Discharge status	home
DRG	09C05V subtotal mastectomies
	without comorbidities
…	…
Principal diagnosis	C504 : Malignant neoplasm of breast,
	Upper-outer quadrant of breast
Related diagnosis	NA
Comorbidities	I10 : Essential (primary) hypertension
Procedures	QEFA004 : Lumpectomy
	

A national scale of costs per DRG for hospitals is computed each year, by measuring costs of stays in about 50 hospitals. Since 2001, an anonymised patient identifier makes it possible to link all the stays of a given patient in different hospitals.

### Building the trajectory of care

We used data from the National PMSI database including all hospitalizations in public or private hospitals for years 2008 to 2010. Data were obtained after approval from the CNIL, the so-called French Data Protection Authority.

**Figure 2:**
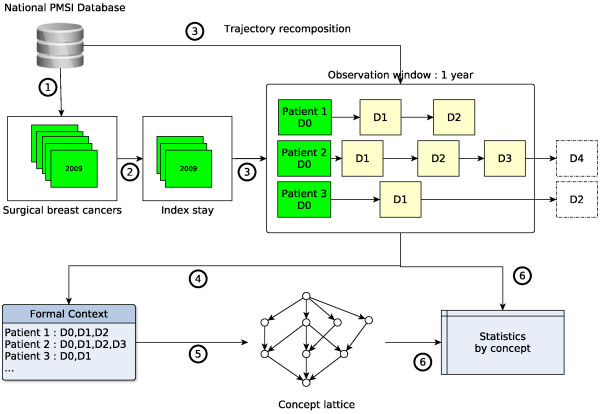
Data processing steps.

Data processing steps are described on Figure 2. For the first step (1), all stays for surgical breast cancer treatment in 2009 were identified. A stay was selected by the co-occurrence of an ICD-10 breast cancer code (C50* or D05*) and a CCAM code of breast surgery. For a given patient, the first identified stay in 2009 was considered as the index stay (setp 2). We looked for previous similar situation in 2008 to check that this index stay was indeed a as far as possible a “new case”. Step 3 consisted in building the one year care trajectory for each patient identified at step 2. This trajectory is defined as the sequence of hospitalizations beginning less than 366 days after the index stay, which is the first element of the trajectory. Hence, the observation window covers any stay, for any health condition, occuring within one year from the index stay. However, the PMSI does not apply to ambulatory radiotherapy session in private facilities, though they represent nearly half of the settings in France. We therefore removed all the ambulatory radiotherapy sessions from the analysis.

Several indicators were recorded for each patient: hospitalization costs, number of stays, cumulative length of stay, number of chemotherapy sessions, death. Hospitalization costs were computed using the national scale of costs for public hospitals. Death could only be identified if happening at hospital using the discharge status.

### Conceptual clustering of trajectories of care

All the principal and related diagnoses codes in the trajectories were used to build a formal context having patients as objects and diagnoses as attributes (step 4). An excerpt of this context is shown in Table 3. For granularity and tractability reasons, only the first 3 digits of codes were used, except for Z-codes from the chapter XXI of the ICD-10 as 4 and 5 digits convey interesting information (see Additional file 1). A concept lattice was then built using the Coron System (available at http://coron.loria.fr) (step 5). In that lattice, each concept intent can be seen as a condition profile of the patients in the corresponding extent.

**Table 3:** An excerpt of the trajectories formal context

**Objects**	**Attribute list**
**(Patient IDs)**	**(Diagnosis codes)**
1	C50, Z768
2	C50
3	C50, R02, Z511
4	C50, I80, Z511
…	…
57552	D05

The resulting lattice holds all possible combinations of diagnoses that are really observed in the patient trajectories. In order to be interpreted, the lattice is filtered according to concepts support and proportion of own objects. Unfrequent or unstable concepts are removed and the remaining ones are manually reviewed by medical experts.

For each concept, summary statistics are computed considering the patients in its extent: death rates, mean hospitalization costs, mean number of stays, mean cumulative length of stay, mean number of chemotherapy sessions, gender frequencies (step 6).

Though the complete concept lattice can produce non-overlapping classes, filtering the most interesting concepts leads to non-disjoint classes. Actually, a patient appearing in a given concept counts for the computation of statistics in all of its superconcepts. Meanwhile, if necessary, FCA can be combined with other techniques to produce disjoint groups. In this work, we used a regression tree analysis to explain the effects of each profile on the total care cost [32]. In this analysis, each concept is considered as a predictor : its value is set to TRUE if the patient appears in the concept extent. A tree was then grown using the R package rpart [33,34] and was evaluated using 10-fold cross-validation to estimate the Mean Square prediction Error (MSE).

## Results

57,552 patients were identified by the selection algorithm. The formal context had 1032 attributes and the resulting lattice had 9,159 concepts. The most frequent and most stable concepts were kept (support ≥ 300 and own objects proportion ≥ 0.1). Table 4 shows the remaining concepts and their related statistics. The most frequent is the concept with an empty intent. This concept holds all the 57,552 patients meaning that no diagnosis code was common to the entire population of the study. This concept can be taken as a baseline for comparison with other morbidity profiles. Its mean cost is 9,600€, including 3,090€ for chemotherapy sessions. The mean number of stays is 2.0 for a mean cumulative length of stay of 7.3 days. Patients had a mean of 2.9 chemotherapy sessions. They were women in 99% of cases with a mean age of 60.4 years.

**Table 4:** Statistics by concepts

**Intent**	**Patients**	**Cost**		**Stays**		**Chemotherapy sessions**	**Death rate**	**Age**
	**n**	**€**	**n**	**Cum. length**	**n**	**Cost**	**%**	
D05	5034	6957	1.9	5.9	0.6	669	0	57.6
C50, H25	517	9499	3.1	8.2	1.1	1165	1	75.9
*∅*	**57552**	**9600**	**2.0**	**7.3**	**2.9**	**3090**	**1**	**60.4**
C50	53535	9902	2.0	7.5	3.1	3318	1	60.6
D05, Z421	482	11471	3.1	11.8	0.3	306	0	50.4
C50, D05	1017	12384	2.8	9.5	3.0	3109	1	56.0
C50, Z421	1339	13484	3.2	12.3	2.0	2055	0	51.2
C50, N61	445	15362	3.5	15.3	3.5	3737	1	60.7
C50, Z452, Z511	13214	15736	2.9	7.8	7.9	8341	1	56.1
C50, Z511	20820	16113	2.7	8.4	8.1	8531	1	55.3
C50, C77	863	16590	3.0	9.6	5.9	6266	1	57.6
C50, C77, Z452, Z511	348	17803	3.5	9.4	7.4	7822	1	57.0
C50, D05, Z511	350	18039	3.1	9.7	8.6	9034	1	53.4
C50, C77, Z511	687	18351	3.1	9.6	7.5	7871	1	55.7
C50, Z421, Z511	332	19946	3.6	12.4	7.9	8288	1	48.5
C50, D61, Z452, Z511	420	22319	4.5	15.5	8.1	8531	3	57.4
C50, D61, Z511	622	22598	4.4	16.4	8.0	8396	3	56.6
C50, C79	372	23052	4.0	28.6	6.3	6587	24	58.9
C50, Z515	365	26139	4.5	43.2	4.2	4371	69	65.5

Statistics are computed on a per patient basis.

53,535 patients had a code of invasive breast cancer (C50) and 5,034 had a code of in situ carcinoma of the breast (D05). The concept (C50, D50) show that, for 1,017 patients, both in situ and invasive neoplasms codes were recorded. The highest cost, 26,139€, was observed for the concept (C50, Z515) coding for invasive neoplasm and palliative care. This concept has also the highest death rate (0.69), number of stays (4.5) and length of stay (43.2 days). The lowest cost, 6,957€, corresponds to the concept of in situ carcinomas of the breast (D05). This concept is associated with the lowest number of stays, length of stay and death rate. However, the concept (C50, D05, Z511) indicates that 371 of these patients had also chemotherapy sessions. Moreover, that group is associated with the highest costs of chemotherapy sessions in Table 4.

Patients in concepts with a code of plastic surgery of breast (Z421) were generally younger: 50.4 years for concept (D05, Z421) and 51.2 for (C50, Z421). At the opposite, senile cataract was observed for older patients: 75.9 years for concept (C50, H25). It can be noticed that the cost for this concept (9,499) is slightly lower than the baseline (9,600) but with a higher number of stays (3.1 vs. 2.0).

Concepts holding patients with advanced malignancies such as secondary locations of lymph nodes (C77), or other and unspecified sites (C79) were associated with higher costs and death rates.

The concept (C50, D61, Z511) is a subconcept of (C50, Z511): all the patients in its extension appear also in the extension of (C50, D61). Comparing the costs of these two concepts reveals that aplastic anaemia (D61) is related with an increase of at least 6400€. The hierarchical structure of the lattice, with super/sub concept relation, allows for such comparisons; for example, N61 (Inflammatory disorders of breast) is associated with increased costs (15,362 vs 9,902) and number of stays (3.5 vs. 2.0) when comparing concepts (C50, N61) with (C50).

**Figure 3:**
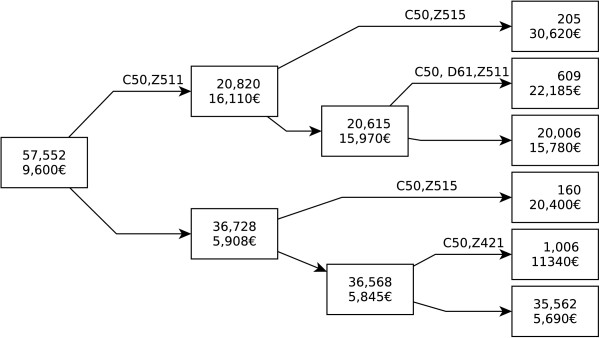
Regression tree.

**Figure 4:**
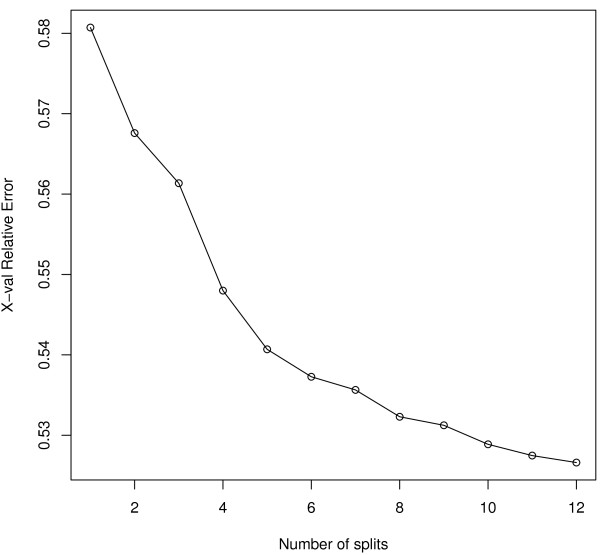
Reduction of the MSE according to the number of splits.

The patients appearing in the extent of a concept also appear in the extents of all its superconcepts. Because of this, a filtered lattice can not be seen as hierarchy of disjoint classes. Whenever it may be desirable to achieve a partition of the population, the lattice can be used in conjunction with other techniques. Figure 3 shows the results of a regression tree where the dependent variable is the cost and the predictors are the concepts in which trajectories of care lie (found in Table 4). Each node is labeled by: 1-the number of patients in the node, 2-the average cost for the node. Arrows are labelled by concept membership. By convention, patients having the profile represented by a concept are in the upper branch of a subtree. Figure 4 shows the relative reduction of the prediction error according to the number of splits. The first split explains most of the cost variability. After 5 splits, giving the tree in Figure 3, additional predictors have little effect on the accuracy of the model.

The first split is given by the concept (C50, Z511): invasive BC and chemotherapy session. 20,820 patients shared these two codes with a mean cost of 16,110€. For other patients (n=36,728), at least one of these two codes had never been used as principal diagnosis. This was associated with a nearly 3 times lower total cost: 5,908€. The other nodes show profiles associated with increased costs: plastic surgery of the breast (Z421), palliative care (Z515) and D61 (aplastic anaemia). Terminal nodes indicate that the majority of the patients (n=35,562, 62%) are associated with the lowest costs (5,690€). Theses patients either had an in situ carcinoma, or had an invasive cancer but no stay for chemotherapy session. They did not have any stay for plastic surgery nor palliative care. The next most frequent profile is the one of patients with invasive cancer and chemotherapy sessions (n=20,006, 35%) for a mean cost of 15,780€.

## Discussion

We have presented an approach aiming at clustering care trajectories and analyzing hospitalization costs according to different morbidity profiles. Our method makes use of existing data from the French national casemix system. This has several advantages. First, there is no additional collection of data. Second, this guaranties a form of consistency, quality, homogeneity of data; like other DRG systems, the PMSI is covered by an official guide of data coding, data recording and data transmission rules [35]. Third, this also ensures stability for long term analysis and ulterior comparisons.

Formal Concept Analysis is a powerful conceptual clustering method. Its implementation is fairly simple and requires only a binary table relating individuals and their attributes. It is able to deal with massive amounts of data and is fully unsupervised, thus requiring no labeled training data. In our study, FCA has produced clinically meaningful categories based on sets of diagnoses. These categories are the result of frequent co-occurrences of ICD 10 codes in the patients care trajectories. In this, FCA can be considered as a “white box” model with a straightforward interpretation. We represented diagnoses by their 3-digit ICD codes. A finer grained representation would have resulted in over specific and small categories, sensible to instability of coding. The level of granularity chosen for the attributes is to be taken in consideration to achieve a compromise between generality and specificity of the concepts. As an interesting side effect, FCA can also be used to assess quality of data in the DRG system. We discovered that 1017 patients had both D05 (in situ) and C50 (invasive) breast cancer codes. A closer look at the data revealed that the main diagnosis at inclusion was C50 for 449 (44%) of them, meaning that D05 was recorded subsequently. It is likely that there are coding errors, though we have no evidence at this time to distinguish them from multiple tumors. The concept (C50, D05) could also reflect genuine diagnosis errors. Besides the potential clinical consequences of such errors, our work suggests that they have an economical impact: the cost of concept (C50, D05, Z511) shows a difference of 1,926€ compared to concept (C50, Z511). This kind of fortuitous discovery is actually one of the goals and one of the benefits of data driven approaches.

We analyzed care trajectories in the field of breast cancer. Since 2001, the French casemix information system uses an anonymized identifier linking all the contacts of a given patient with the healthcare system. It is now possible to study groups of patients at a national scale and to analyze resource use in an quasi-exhaustive manner. Thus, our results are not submitted to sampling error. However, they strongly depend on the algorithm used to select patients and build the care trajectory. Several studies have assessed the use of administrative databases to detect cancer cases [12,36,37]. Our method selects patients with surgical breast cancer on a combination of diagnosis and procedure codes which can reduce the false positive rate [38]. The constitution of the care trajectory should be elaborated in order to minimize censoring bias. We standardized the observation window by fixing the start of the trajectory as the first stay for breast cancer surgery and recording all the stays occuring within one year, a sufficient follow-up time to capture the first phase of treatment. The events constituting the care trajectory were any stay recorded by the PMSI, with the exception of ambulatory radiotherapy sessions. This limit in our study is due to the absence of recording of radiotherapy by the PMSI in private settings.

Costs were estimated using the national scale of costs per DRG in hospitals and reflect use of resources by the care providers. Our results show a great variability of costs according to morbidity profiles. Our approach may be used by care providers to take strategic decisions and adapt their resources from a patient-centric point of view, taking into account a whole trajectory rather than a single acute episode of care. Depending on their capacities, they could either evolve towards a more integrated approach, or on the contrary, take advantage of their core competencies and position themselves in the continuum of chronic care. Our method brings also valuable information for hospitals taking part in repetitive treatments (for example annual costs of care per patient, number and total length of episodes of care). Such information is generally unknown from hospital managers because hospital information systems are not aware of what is happening outside the facility in a trajectory of care. At a regional level, this information may help hospital managers to better plan recruitment of patients according to local needs and other hospitals activity. Moreover, as the French system is incited by authorities to develop multidisciplinarity and collaborations, the analysis of care trajectories is essential for implementing more integrated care processes. This work shows how administrative databases and data mining methods can be used to produce descriptions of care trajectories that are both medical and economical. Our approach can highlight discussions and support decisions between partners who are setting up collaborations.

From the institutional standpoint, our method allows health policy makers to set-up cost-of-illness analysis on a national scale. Compared to other patient management systems such as Ambulatory Care Groups [39], it is flexible and reuses existing data, avoiding the drawbacks of a dedicated information system. Though it is not completely disconnected from the DRGs, our grouping of care trajectories is at first unsupervised and aggregates similar conditions profiles. It can be used in combination with other supervised techniques such as recursive partitioning [32], frequently used in DRG systems [40], to explain costs of care. Analysis of care trajectories could also be used for fair allocation of resources and funding. Indeed, fee-for-service may introduce imbalance funding between the different parts of some trajectories. A global vision of the trajectory can be necessary to redistribute and equilibrate resources between the healthcare providers through prices adjustment, while keeping the overall care expenditure constant.

Our approach is limited by the availability of linkable data. In France, an anonymized identifier can track a patient along its journey through the healthcare system at a national scale. Even though health administrative databases are implemented in many countries, such an identifier may not always exist or may have a limited coverage of the population. Our study focuses on breast cancer but other chronic pathologies can be considered. However, for many of them, care is delivered on an ambulatory basis. In that context, the availability of diagnosis data can be reduced. Eventually, the identification of cases may be subjected to misclassification bias [41,42].

## Conclusion

In this paper, we have presented an approach for clustering trajectories of care. Our system is based on Formal Concept Analysis and reuses routinely available claim data. It is flexible and facilitates the longitudinal exploration of treatment practices in the field of chronic diseases. With the example of breast cancer in France, we have demonstrated the possibility of studying trajectories of care at a national level and describing hospitalizations costs according to condition profiles. Classes resulting from an unsupervised process were clinically and economically relevant. Our approach could help healthcare professionals and policy makers to setup cost-of-illness analysis and plan allocation of resources on a patient basis rather than a visit basis.

## Competing interests

The authors declare that they have no competing interests.

## Authors’ contributions

NJ formulated the study design, performed data processing and analysis, interpreted the results and drafted the manuscript. GN performed data pre-processing. GN and CQ participated in the study design and interpretation of results. All authors participated in revising the manuscript. All authors read and approved the final manuscript.

## Pre-publication history

The pre-publication history for this paper can be accessed here:

http://www.biomedcentral.com/1472-6947/13/130/prepub

## Supplementary Material

Additional file 1 **ICD10 codes used in the article.** An CSV file with ICD10 codes and their labels. Click here for file
